# Editorial: Gut microbiota changes: a key driver of increased food allergy prevalence

**DOI:** 10.3389/falgy.2026.1787825

**Published:** 2026-02-02

**Authors:** Ibrahim Musa, Maurizio Mennini

**Affiliations:** 1 Department of Pathology Microbiology Immunology, New York Medical College, Valhalla, NY, United States; 2 Universita Degli Studi di Roma La Sapienza Dipartimento di Neuroscienze Salute Mentale e Organi di Senso, Rome, Italy

**Keywords:** bacterial enzymes, cross reactivity, food allergy, food allergy therapeutics, gut microbiota, probiotics

Food allergy (FA) continues to rise globally, particularly in industrialized countries, representing a growing public health challenge that disproportionately affects children. The burden extends beyond medical management to impact families’ quality of life and healthcare systems.

Increasing evidence highlights the gut microbiota as a critical regulator of mucosal immune homeostasis and tolerance to dietary antigens.

Dysbiosis, an imbalance between protective and pro-inflammatory microbial taxa, has emerged as a central mechanism linking environmental exposures, epithelial barrier dysfunction, and aberrant immune responses.

This *Research Topic* brings together four complementary reviews that collectively elucidate how gut microbial alterations may drive the increasing prevalence of FA and, importantly, how this knowledge can inform novel preventive and therapeutic approaches.

## Microbiome signatures in pediatric food allergy

In their comprehensive scoping review, Farnetano et al. examined 34 studies describing gut microbiota composition in children with food allergy compared to healthy controls. The authors identified a consistent pattern of reduced beneficial taxa—notably *Bifidobacteriaceae* and *Lactobacillaceae*—and increased abundance of potentially pathogenic species, including *Enterobacteriaceae*, *Clostridium sensu stricto*, and *Ruminococcus gnavus*. These microbial imbalances were further linked to environmental modifiers acting during the first 1,000 days of life, such as antibiotic exposure, cesarean delivery, formula feeding, and ultra-processed food consumption.

The review underscores how these early-life exposures alter microbial metabolites, especially short-chain fatty acids (SCFAs) like butyrate, which are pivotal for the expansion of regulatory T cells (Tregs) and maintenance of oral tolerance. Farnetano and colleagues propose that preserving or restoring microbial diversity during critical developmental windows could represent a key strategy in FA prevention.

## Barrier integrity and immune–microbial dialogue

Valitutti et al. provided an integrative overview of how intestinal permeability, dietary antigens, and microbiota interact to influence immune homeostasis. The gut barrier, comprising the microbiota, mucus, epithelium, and mucosal immunity, acts as a selective interface between external antigens and the host. Its disruption, often triggered by westernized diets, pollution, infections, or antibiotics, can lead to uncontrolled antigen translocation and inflammation.

The review describes how microbial products such as SCFAs reinforce tight-junction integrity and regulate epithelial cytokine responses, whereas dysbiosis and loss of beneficial species may increase the expression of pore-forming proteins like claudin-2, thereby favoring a “leaky gut.” This permeability facilitates antigen access to the lamina propria, amplifying Th2 polarization and IgE production. Importantly, Valitutti and colleagues highlight that probiotic strains, including *Lactobacillus reuteri* and *Bifidobacterium lactis*, can mitigate barrier dysfunction in experimental models, opening new translational perspectives for FA management.

## Microbiota and cross-reactivity: a new frontier

In their mini-review, Taico Oliva et al. explored the interplay between the gut microbiome and cross-reactivity among food allergens. Traditionally, cross-reactivity has been explained by shared amino acid sequences or conformational epitopes between different allergens. However, the authors emphasize that microbial diversity and metabolites profoundly modulate these immune interactions.

They describe how dysbiosis-induced intestinal permeability can facilitate the entry of structurally similar microbial and dietary antigens, intensifying IgE-mediated responses through mechanisms of molecular mimicry. Beneficial microbes such as *Lactobacillus* and *Bifidobacterium*, through production of SCFAs and promotion of Tregs and IgA, help maintain immune tolerance and may reduce cross-sensitization. This review introduces a conceptual shift: microbial ecology not only shapes sensitization to primary allergens but may also determine whether clinically relevant cross-reactivity develops.

## Therapeutic modulation: probiotics and adjuvants in immunotherapy

Mennini et al. examined how manipulating the microbiota could enhance outcomes of food allergen-specific immunotherapy (FA-AIT). While oral, sublingual, and epicutaneous immunotherapies have shown promise in achieving desensitization and sustained unresponsiveness, variability in long-term efficacy and safety remains a concern.

The review highlights probiotics, particularly *Lactobacillus rhamnosus*, as potential adjuvants that promote Treg differentiation, suppress Th2 cytokines (IL-4, IL-5), and boost IL-10 and TGF-β production. Clinical evidence, such as the use of *L. rhamnosus* combined with peanut oral immunotherapy, demonstrates higher sustained unresponsiveness rates and improved immune profiles compared to placebo. The authors also discuss emerging adjuvant strategies, including biologics, nanoparticles, and herbal compounds like FAHF-2, which may enhance allergen delivery and safety. Integrating probiotics and other adjuvants into AIT protocols could thus transform FA-AIT into a safer, more durable, and mechanistically grounded therapy.

## Integrative insights and future perspectives

Across these contributions, a cohesive picture emerges: food allergy pathogenesis results from the convergence of dysbiosis, barrier dysfunction, and immune miscommunication.

The reviewed evidence emphasizes three key concepts:

1. Timing and windows of susceptibility: The early-life period is critical for establishing balanced microbial colonization and immune tolerance. Preventive strategies should focus on maternal health, delivery mode, feeding practices, and prudent antibiotic use.
2. Barrier health as a therapeutic target: Restoring epithelial integrity through diet, probiotics, or microbiota-derived metabolites can reduce antigen penetration and modulate Th2-driven inflammation.
3. Microbiota-informed therapies: Rationally selected microbial consortia or postbiotic formulations may complement immunotherapy and dietary interventions, offering personalized, mechanism-based approaches.

Clinically, these insights encourage pediatricians and allergists to consider microbiome preservation and modulation as integral components of FA prevention and care. However, translation from association to causation remains a major research frontier. Future studies should incorporate longitudinal designs, functional metagenomics, and standardized endpoints, such as sustained unresponsiveness, to validate microbiota-targeted interventions.

## Concluding remarks

This *Research Topic* underscores the central role of the gut microbiota as both a driver and a modifiable determinant of food allergy. By integrating knowledge from pediatric dysbiosis patterns, epithelial biology, antigen cross-reactivity, and microbiome-based therapeutics, the collected works chart a pathway from mechanistic understanding to clinical innovation. We hope these contributions will inspire multidisciplinary collaborations aimed at developing precision, microbiome-informed strategies for the prevention and treatment of food allergy.

